# 减低强度预处理单份脐带血移植治疗重型再生障碍性贫血的临床研究

**DOI:** 10.3760/cma.j.issn.0253-2727.121090-20230928-00146

**Published:** 2024-01

**Authors:** 月 吴, 宝林 汤, 闿迪 宋, 光宇 孙, 田中 潘, 爱杰 黄, 冰冰 颜, 小玉 朱

**Affiliations:** 1 中国科学技术大学附属第一医院（安徽省立医院）血液内科，合肥 230001 Department of Hematology, the First Affiliated Hospital of USTC, Division of Life Sciences and Medicine, University of Science and Technology of China, Hefei 230001, China; 2 血细胞研究及应用安徽省重点实验室，合肥 230001 Anhui Provincial Key Laboratory of Blood Research and Applications, Hefei 230001, China; 3 中国科学技术大学生命科学与医学部，血液和细胞治疗研究所，合肥 230001 Blood and Cell Therapy Institute, Division of Life Sciences and Medicine, University of Science and Technology of China, Hefei 230001, China

**Keywords:** 重型再生障碍性贫血, 抗人胸腺细胞球蛋白, 减低强度预处理, 脐带血, Severe aplastic anemia, Antithymocyte globulin, Reduced-intensity conditioning, RIC, Umbilical cord blood

## Abstract

**目的:**

评价减低强度预处理（RIC）方案单份非血缘脐带血移植（sUCBT）治疗重型再生障碍性贫血（SAA）的临床疗效。

**方法:**

纳入2021年1月至2023年7月在中国科学技术大学附属第一医院（安徽省立医院）血液科接受sUCBT的63例SAA患者，对其临床资料进行回顾性分析。移植预处理均采用RIC方案：全身放射治疗（TBI）/全骨髓照射（TMI）4 Gy+氟达拉滨（Flu）（总量200 mg/m^2^，分5 d给药）+环磷酰胺（Cy）（总量120 mg/kg，分2 d给药），其中11例患者在上述预处理方案基础上加用兔抗人胸腺细胞免疫球蛋白（rATG）2 mg/kg（rATG组）。以环孢素A（CsA）联合霉酚酸酯（MMF）方案预防移植物抗宿主病（GVHD）。

**结果:**

rATG组与非rATG组患者一般资料及移植物情况差异均无统计学意义（*P*>0.05）。rATG组所有患者均获得造血重建，非rATG组5例患者发生原发性植入失败。两组移植后42 d中性粒细胞累积植入率及60 d血小板累积植入率差异无统计学意义。rATG组Ⅱ～Ⅳ度急性GVHD发生率低于非rATG组［10.0％（95％*CI* 0.5％～37.4％）对46.2％（95％ *CI* 32.1％～59.1％），*P*＝0.032］，两组Ⅲ/Ⅳ度急性GVHD及慢性GVHD发生率差异均无统计学意义（*P*＝0.428，*P*＝0.107）。两组植入前综合征（PES）、血流感染、巨细胞病毒（CMV）血症及出血性膀胱炎发生率差异均无统计学意义（*P*>0.05）。存活患者中位随访时间为536（61～993）d，全部患者移植后1年移植相关死亡率（TRM）为13.0％（95％*CI* 6.7％～24.3％），非rATG组、rATG组分别为15.5％（95％ *CI* 8.1％～28.6％）、0％（*P*＝0.189）。全部患者移植后1年总生存（OS）率为87.0％（95％*CI* 75.7％～93.3％）。rATG组、非rATG组移植后1年OS率分别为100％、84.5％（95％*CI* 71.4％～91.9％）（*P*＝0.198）。

**结论:**

含TBI或TMI联合Flu/Cy的RIC方案sUCBT治疗SAA具有较好的疗效。早期应用小剂量rATG可降低移植后急性GVHD发生率，未增加植入失败及感染风险。

## Abstract (en)

**Objective:**

To evaluated the clinical efficacy of a reduced-intensity preconditioning regimen for single non-blood-related umbilical cord blood transplantation（sUCBT）in the treatment of severe aplastic anemia（SAA）.

**Methods:**

The clinical data of 63 patients with SAA who underwent sUCBT from January 2021 to July 2023 at the Department of Hematology of the First Affiliated Hospital of USTC were retrospectively analyzed. Fifty-two patients received total body irradiation/total bone marrow irradiation（TMI）combined with fludarabine or a cyclophosphamide- conditioning regimen（non-rATG group）, while 11 patients received rabbit anti-human thymocyte immunoglobulin（rATG）combined with TMI, fludarabine, or the cyclophosphamide-conditioning regimen（rATG group）. All patients received cyclosporine A and mycophenolate mofetil for graft-versus-host disease（GVHD）prophylaxis. Complications post-transplantation and long-term survival were compared between the two groups.

**Results:**

The baseline parameters were balanced between the two groups（*P*>0.05）. In the rATG group, all patients achieved stem cell engraftment, and in the non-rATG group, five patients had primary graft failure. There was no significant difference in the cumulative incidence of neutrophil engraftment at 42 days after transplantation or platelet engraftment at 60 days between the two groups. The incidence of grade Ⅱ-Ⅳ acute GVHD in the rATG group was significantly lower than in the non-rATG group（10.0％ *vs*. 46.2％, *P*＝0.032）, and the differences in the cumulative incidences of grade Ⅲ/Ⅳ acute GVHD and 1-year chronic GVHD were not statistically significant（*P*＝0.367 and *P*＝0.053, respectively）. There were no significant differences in the incidences of pre-engraftment syndrome, bacterial bloodstream infections, cytomegalovirus viremia, or hemorrhagic cystitis between the two groups（*P*>0.05 for all）. The median follow-up time for surviving patients was 536（61-993）days, and the 1-year transplantation related mortality（TRM）of all patients after transplantation was 13.0％（95％ *CI* 6.7％–24.3％）. Among the patients in the non-rATG and rATG groups, 15.5％（95％ *CI* 8.1％–28.6％）and 0％（*P*＝0.189）, respectively, had mutations. The 1-year overall survival（OS）rate of all patients after transplantation was 87.0％（95％ *CI* 75.7％–93.3％）. The 1-year OS rates in the rATG group and non-rATG group after transplantation were 100％ and 84.5％, respectively（95％ *CI* 71.4％–91.9％）（*P*＝0.198）.

**Conclusion:**

The preliminary results of sUCBT with a low-dose irradiation-based reduced-intensity conditioning regimen with fludarabine/cyclophosphamide for the treatment of patients with SAA showed good efficacy. Early application of low-dose rATG can reduce the incidence of acute GVHD after transplantation without increasing the risk of implantation failure or infection.

重型再生障碍性贫血（SAA）是一类起病急、发病重、死亡率高、需尽早接受治疗的骨髓衰竭性疾病，其主要治疗方法包括异基因造血干细胞移植（allo-HSCT）和抗人胸腺细胞球蛋白（ATG）联合环孢素A（CsA）的免疫抑制治疗（IST）。同胞全相合供者造血干细胞移植（MSD-HSCT）或单倍体造血干细胞移植（haplo-HSCT）是≤50岁SAA患者的一线治疗选择[1]，而对于>50岁、缺乏全相合同胞供者或IST失败的患者，无关供者造血干细胞移植（UD-HSCT）可显著改善此类患者预后。非血缘脐带血因获取方便快捷、免疫原性低、移植后慢性移植物抗宿主病（GVHD）发生率低等优点而成为理想的造血干细胞来源。随着高分辨HLA配型的普及以及支持治疗的不断进步，非血缘脐带血移植（UCBT）治疗SAA的长期生存率也在不断提高，但对于大体重、成年SAA患者，脐带血干细胞含量不足而造成的植入失败和移植后急性GVHD仍是影响患者早期生存的重要因素[2]–[3]。本中心采用减低强度预处理（RIC）非血缘脐带血移植（sUCBT）治疗63例SAA患者获得比较理想的疗效。

## 病例与方法

一、病例

本研究连续性纳入于2021年1月至2023年7月于中国科学技术大学附属第一医院（安徽省立医院）血液科接受sUCBT的63例获得性SAA患者。所有患者及其家属均在移植前签署知情同意书。

二、脐带血的选择

根据我中心标准[4]选择脐带血，脐带血和患者的 HLA 配型采用10个HLA-A、-B、-C、-DRB1和-DQB1位点高分辨基因分型。脐带血与患者低分辨HLA相合度≥4/6，高分辨HLA相合度≥5/10。移植前1个月内患者进行抗HLA抗体检测，如患者体内存在抗目标脐带血任一位点抗体且平均荧光强度（MFI）≥500，视为DSA阳性，该份脐带血不予采用。目标脐带血进行小管复苏后，每份脐带血总有核细胞（TNC）≥2.0×10^7^/kg（患者体重）和CD34^+^细胞≥1.0×10^5^/kg（患者体重）。

三、预处理方案及GVHD预防

所有患者采用氟达拉滨（Flu）-环磷酰胺（Cy）-全身照射（TBI）/全骨髓照射（TMI）的RIC方案：Flu 40 mg/m^2^每日1次，−6 d～−2 d；Cy 60 mg/kg每日1次，−3 d、−2 d；TBI或TMI总剂量为400 cGy，−1 d；11例患者在上述方案基础上加用兔抗人胸腺细胞免疫球蛋白（rATG）2 mg/kg（−7 d给药），移植前存在活动性感染患者避免加用rATG。全部患者采用环孢素A（CsA）联合短程霉酚酸酯（MMF）方案[5]预防GVHD。其他支持治疗按照本中心移植常规方案[6]进行。

四、疗效评定及随访

主要研究终点为移植后42 d中性粒细胞累积植入率和移植后Ⅱ～Ⅳ度急性GVHD发生率。急性GVHD的诊断和分级采用西雅图标准[7]，慢性GVHD的诊断和分级采用2014年NIH标准[8]。采用查阅病历、电话联系等方式进行随访，随访截止日期为2023年9月25日。

五、统计学处理

连续变量比较采用Student's *t*检验或Mann-Whitney检验。分类变量采用Chi-square检验或Fisher's精确检验。中性粒细胞累积植入率、血小板累积植入率、急性GVHD累积发生率、慢性GVHD累积发生率采用含竞争风险的Gray's检验，中性粒细胞累积植入率及血小板累积植入率将死亡视为竞争事件，急性及慢性GVHD累积发生率将死亡、原发性植入失败视为竞争事件[9]。预计总生存（OS）率采用Kaplan-Meier法计算，组间比较采用Log-rank检验。统计分析应用R 4.2.2软件，*P*<0.05为差异有统计学意义。

## 结果

一、病例特征

所有患者符合SAA的诊断标准[1]，其中6例患者发病时外周血中性粒细胞绝对值（ANC）小于0.2×10^9^/L，诊断为极重型再生障碍性贫血（VSAA）。新诊断患者9例（14.3％），IST治疗失败54例（85.7％）。63例患者均接受Flu/Cy/低剂量TBI（15例）或TMI（48例）的RIC方案，根据是否加用rATG分为rATG组（11例）和非rATG组（52例）。两组患者性别、年龄、体重、输注TNC及CD34^+^细胞输注量、供患者HLA相合度等特征差异均无统计学意义（*P*>0.05），详见表1。所有存活患者中位随访时间为536（61～993）d，rATG组患者中位随访时间短于非rATG组患者（310 d对704 d，*P*<0.001）。

二、造血重建

全部63例患者中，5例发生原发性植入失败，均在非rATG组，其中4例行挽救性单倍体造血干细胞移植获得长期生存，1例于移植后56 d死于多脏器功能衰竭。移植后42 d中性粒细胞累积植入率为92.1％（95％ *CI* 83.8％～97.1％），移植后60 d血小板累积植入率为66.7％（95％ *CI* 53.4％～77.0％）。非rATG组、rATG组中性粒细胞中位植入时间分别为16（12～27）d、16（12～22）d（*P*＝0.819），移植后42 d中性粒细胞累积植入率分别为90.4％（95％*CI* 80.6％～96.5％）、100％（*P*＝0.172），血小板中位植入时间分别为40（20～147）d、33（24～64）d（*P*＝0.712），移植后60 d血小板累积植入率分别为65.4％（95％*CI* 50.5％～76.8％）、72.7％（95％*CI* 31.5％～91.6％）（*P*＝0.563）。

**表1:** 63例接受减低强度预处理单份脐带血移植重型再生障碍性贫血患者的临床资料

临床特征	总体（63例）	非rATG组（52例）	rATG组（11例）	*P* 值
男性［例（%）］	37（58.7）	29（55.8）	8（72.7）	0.483
移植时年龄［岁，*M*（范围）］	11（1~43）	11（1~39）	11（3~43）	0.856
移植时体重［kg，*M*（范围）］	42（10~101）	41（10~101）	43（13~76）	0.814
冷冻前总有核细胞数［×10^7^/kg，*M*（范围）］	14.04（6.24~22.97）	14.27（6.24~22.97）	11.98（8.86~22.02）	0.190
总有核细胞输注量［×10^7^/kg，*M*（范围）］	3.02（0.97~9.21）	3.02（0.97~9.21）	3.04（1.32~8.84）	0.754
冷冻前CD34^+^细胞数［×10^5^/kg，*M*（范围）］	7.49（2.28~31.31）	7.54（2.28~31.31）	7.30（4.24~10.07）	0.752
CD34^+^细胞输注量［×10^5^/kg，*M*（范围）］	2.50（0.39~11.60）	2.50（0.39~11.60）	2.95（1.48~8.40）	0.652
抗HLA抗体阳性［例（%）］	22（34.9）	16（30.8）	6（54.5）	0.248
供患者HLA相合位点［例（%）］				0.250
9~10/10	26（41.3）	23（44.2）	3（27.3）	
7~8/10	30（47.6）	23（44.2）	7（63.6）	
≤6/10	7（11.1）	6（11.5）	1（9.1）	
供患者ABO血型匹配［例（%）］				0.982
全相合	25（39.7）	22（42.3）	3（27.3）	
次要不合	17（27.0）	15（28.8）	2（18.2）	
主要不合	15（23.8）	10（19.2）	5（45.5）	
主次不合	6（9.5）	5（9.6）	1（9.1）	
诊断至移植间隔时间［月，*M*（范围）］	19.5（1.2~164.0）	21.0（1.2~164.0）	20.6（1.7~105.7）	0.852
存活患者随访时间［d，*M*（范围）］	536（61~993）	704（138~993）	310（61~476）	<0.001

注 rATG：兔抗人胸腺细胞免疫球蛋白

三、急性和慢性GVHD发生情况

全部患者中，移植后Ⅱ～Ⅳ度、Ⅲ/Ⅳ度急性GVHD发生率分别为40.1％（95％CI 27.9％～52.1％）、19.2％（95％*CI* 10.5％～29.9％）。非rATG组可评估患者47例，24例发生Ⅱ～Ⅳ度急性GVHD，rATG组1例患者移植后44 d因CsA改口服致血药浓度明显下降而发生Ⅳ度肠道急性GVHD。非rATG组Ⅱ～Ⅳ度急性GVHD累积发生率高于rATG组［46.2％（95％ *CI* 32.1％～59.1％）对10.0％（95％*CI* 0.5％～37.4％），*P*＝0.032］（图1），Ⅲ/Ⅳ度急性GVHD累积发生率差别无统计学意义［21.2％（95％ *CI* 11.2％～33.2％）对10.0％（95％*CI* 0.5％～37.4％），*P*＝0.367］。全部患者移植后1年慢性GVHD累积发生率为35.8％（95％*CI* 22.5％～49.3％）。非rATG组19例患者发生慢性GVHD，其中13例为以皮肤或肝功能异常为表现的轻型；rATG组未发生慢性GVHD。两组患者移植后1年慢性GVHD累积发生率分别为37.1％（95％*CI* 23.5％～50.8％）、0％（*P*＝0.053）。

**图1:**
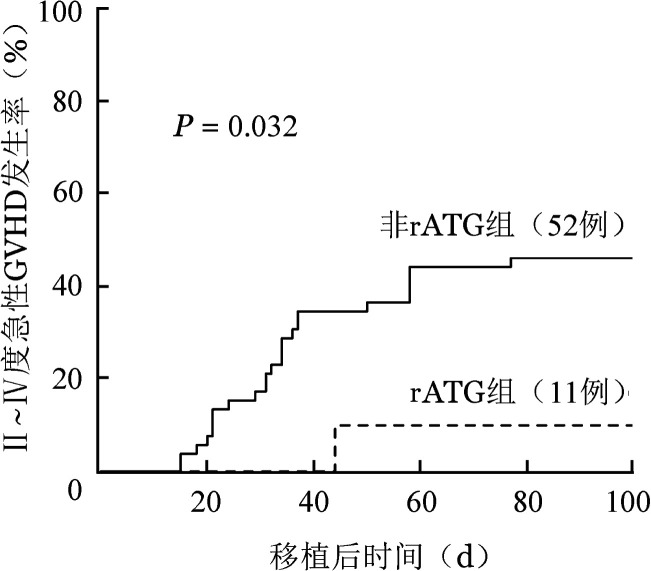
含兔抗人胸腺细胞免疫球蛋白（rATG）预处理对重型再生障碍性贫血患者单份脐带血移植后Ⅱ～Ⅳ度急性移植物抗宿主病（GVHD）发生率的影响

四、移植相关死亡及其他并发症

全部患者移植后1年TRM为13.0％（95％*CI* 6.7％～24.3％），非rATG组、rATG组分别为15.5％（95％ *CI* 8.1％～28.6％）、0％（*P*＝0.189）。rATG组植入前综合征（PES）发生率低于非rATG组（36.4％对67.3％，*P*＝0.086）。两组患者移植后细菌血流感染、CMV血症，出血性膀胱炎发生率差异均无统计学意义（*P*＝1.000，*P*＝0.325，*P*＝0.683）。本研究中无患者发生肝静脉闭塞病（HVOD），rATG组无患者发生血清病。截至随访结束，rATG组未观察到EB病毒再激活、移植后淋巴组织增殖性疾病（PTLD）或第二肿瘤。

五、生存分析

随访至2023年9月25日，非rATG组有8例患者死亡，中位死亡时间为移植后74（50～167）d，rATG组无死亡病例。全部患者移植后1年OS率为87.0％（95％*CI* 75.7％～93.3％）。8例死亡患者中，3例死于重度急性GVHD，1例死于移植相关血栓性微血管病（TA-TMA），2例死于呼吸衰竭，2例死于多脏器功能衰竭。rATG组、非rATG组移植后1年OS率分别为100％、84.5％（95％*CI* 71.4％～91.9％）（*P*＝0.198），OS曲线见图2。

**图2:**
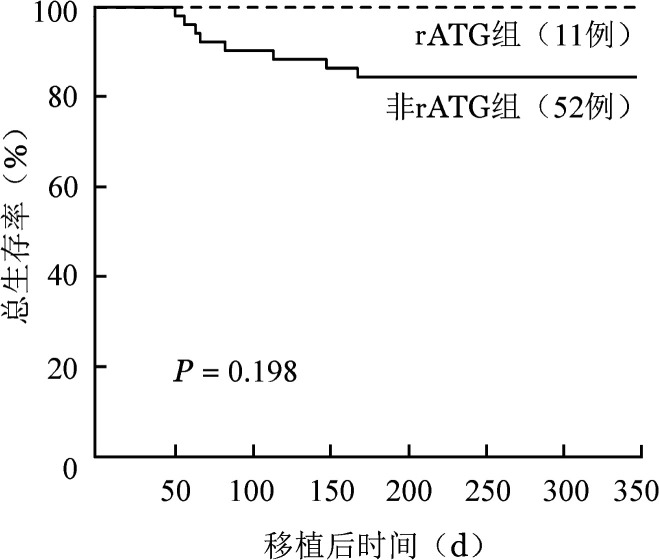
含兔抗人胸腺细胞免疫球蛋白（rATG）预处理对重型再生障碍性贫血患者单份脐带血移植后总生存的影响

## 讨论

植入失败、预处理相关毒性和GVHD是SAA患者进行allo-HSCT的主要障碍。相较于其他UD-HSCT和haplo-HSCT，UCBT因其慢性GVHD发生率低、生活质量较高等优势更适用于不需要移植物抗白血病（GVL）效应的SAA患者。然而，由于造血干、祖细胞数量有限，早期UCBT治疗SAA植入失败率较高，疗效非常有限[10]–[11]。近年来，随着技术的不断改进，UCBT治疗SAA的疗效得到了很大的提高。2018年法国骨髓移植和细胞治疗学会报道了一项前瞻性Ⅱ期临床试验（APCORD研究），26例IST治疗失败的SAA患者接受UCBT，移植后60 d植入率高达88％，1年OS率为88.5％[13]。影响UCBT治疗SAA疗效的两个主要因素是最适脐带血的选择和预处理方案的优化。

最适脐带血的选择是SAA患者进行UCBT治疗成功的第一步。对于骨髓衰竭性疾病脐带血的选择标准，欧洲移植协会推荐供患者进行8个位点（HLA-A、-B、-C和-DRB1）基因型配型，至少HLA 5～8/8相合，冷冻前总有核细胞数（TNC）≥3.5×10^7^/kg；CD34^+^细胞≥1.7×10^5^/kg[12]。日本对脐带血的标准要求较低，可接受TNC≥2.0×10^7^/kg和≥HLA 4/6的HLA-A、-B和-DRB1的抗原配型[13]。日本学者Hiramoto等[14]回顾性分析115例接受sUCBT治疗的再生障碍性贫血（AA）患者，中性粒细胞累积植入率为76.5％，多因素分析发现，冷冻前CD34^+^细胞≥ 0.7×10^5^/kg是促进中性粒细胞植入的独立影响因素。本研究63例患者，仅5例发生原发性植入失败，中性粒细胞累积植入率高达92.1％，明显优于既往研究报道，这可能与本研究中脐带血冷冻前TNC和CD34^+^细胞数均较高且HLA配型采用10个位点基因点分型有关。

预处理方案的优化是决定患者预后的另一重要因素。我中心早期sUCBT治疗SAA的预处理方案采用Flu 120 mg/m^2^+Cy 1200 mg/m^2^+抗人T细胞兔免疫球蛋白30 mg/kg，所有患者脐带血均植入失败，虽造血重建快于既往单用IST治疗患者，但并未解决IST后复发及克隆演变问题[15]。我们认为该方案中低剂量Cy及大剂量人T细胞兔免疫球蛋白可能是导致植入失败的主要原因。2019年美国脐带血移植和细胞治疗协会（ASTCT）提出的GVHD预防指南中[16]，建议在UCBT中避免使用ATG，同时既往多个研究发现大剂量兔源ATG（rATG 7.5～20 mg/kg或抗人T细胞兔免疫球蛋白30～60 mg/kg）可导致感染风险增加和免疫功能重建延迟[17]–[22]。Kudo等[23]报道了RIC方案sUCBT治疗27例SAA患者的临床疗效（11例患者在预处理方案中不加用rATG），非rATG组植入失败发生率明显降低（0％对56％），5年OS率明显提高（100.0％对48.6％）。Hiramoto等[14]报道的研究中，患者接受的预处理方案为TBI（≤4 Gy）联合Flu/Cy或Flu/Mel，结果发现辐照剂量达4 Gy是促进中性粒细胞植入的独立影响因素，且Cy大于100 mg/kg亦明显促进中性粒细胞的植入。本研究中所有患者照射剂量为4 Gy，48例（76.2％）患者采用TMI（4 Gy），相较于TBI可更大限度保护患者的脏器功能。前瞻性APCORD研究中26例SAA患者采用的预处理方案为TBI（2 Gy）/Flu/Cy联合rATG 5 mg/kg，移植后60 d植入率达88％ [15]。本研究中52例患者采用TBI/TMI 4 Gy联合Flu/Cy的RIC方案，移植后中性粒细胞植入率为90.4％，基本解决了UCBT在SAA患者中的低植入率问题。然而，该预处理方案下患者移植后免疫反应发生率较高，因此11例患者在上述方案基础上加用rATG 2 mg/kg，Ⅱ～Ⅳ度急性GVHD发生率低于非rATG组（10.0％对46.2％，*P*＝0.032），rATG组所有患者均获得供者造血干细胞植入。除ATG的使用剂量外，其使用时间也可能对移植疗效产生影响。Laurent等[19]分析了661例成年患者接受UCBT的临床资料，其中82例患者在预处理方案中加用rATG，根据rATG停用时间分组，结果发现rATG停用距干细胞回输时间越短，患者长期生存率越低。本中心于脐带血回输前7 d应用rATG，以期最大限度减少对移植物的影响，提高患者长期生存。

本研究为回顾性研究，患者例数较少，rATG组患者随访时间较短。目前我中心采用含ATG的RIC方案sUCBT治疗SAA的多中心前瞻性临床试验正在招募中（NCT06039436）；其次，指南对于脐带血的选择标准大多数为冷冻前TNC和CD34^+^细胞数，在实际临床中，我们更关注小管复苏后及输注时的细胞数，在该预处理方案背景下对于小管复苏后及输注时的脐带血细胞数推荐还需要进一步研究。

本研究结果初步显示，含TBI或TMI联合Flu/Cy的RIC方案sUCBT治疗SAA患者具有较好的疗效。早期应用小剂量rATG可降低移植后急性GVHD发生率，未增加植入失败及感染风险。以上结论尚需进一步研究验证。
